# Parallel changes in the taxonomical structure of bacterial communities exposed to a similar environmental disturbance

**DOI:** 10.1002/ece3.37

**Published:** 2011-12

**Authors:** Karine Laplante, Nicolas Derome

**Affiliations:** Institut de Biologie Intégrative et des Systèmes (IBIS)1030 rue de la Médecine Université Laval, Québec, Canada

**Keywords:** Applied ecology, bacteria, community ecology, evolutionary ecology

## Abstract

Bacterial communities play a central role in ecosystems, by regulating biogeochemical fluxes. Therefore, understanding how multiple functional interactions between species face environmental perturbations is a major concern in conservation biology. Because bacteria can use several strategies, including horizontal gene transfers (HGT), to cope with rapidly changing environmental conditions, potential decoupling between function and taxonomy makes the use of a given species as a general bioindicator problematic. The present work is a first step to characterize the impact of a recent polymetallic gradient over the taxonomical networks of five lacustrine bacterial communities. Given that evolutionary convergence represents one of the best illustration of natural selection, we focused on a system composed of two pairs of impacted and clean lakes in order to test whether similar perturbation exerts a comparable impact on the taxonomical networks of independent bacterial communities. First, we showed that similar environmental stress drove parallel structural changes at the taxonomic level on two independent bacterial communities. Second, we showed that a long-term exposure to contaminant gradients drove significant taxonomic structure changes within three interconnected bacterial communities. Thus, this model lake system is relevant to characterize the strategies, namely acclimation and/or adaptation, of bacterial communities facing environmental perturbations, such as metal contamination.

## Introduction

Increasing anthropogenic activities accelerate the deterioration of various ecosystems. Because bacterial communities play a central role in regulating biogeochemical fluxes (Xu 2006), there is an urgent need to elucidate the processes allowing them to acclimate or adapt to environmental disturbances and predict their impacts on ecosystem stability. Biodiversity in a dynamic ecosystem provides insurance against the loss of certain species (Folke 1996). Species richness within bacterial communities engenders a corresponding range of behavioral reactions and metabolic pathways. Community level metabolic plasticity facilitates rapid acclimation and adaptation to environmental fluctuations (Armitage et al. 2003). Consequently, bacterial communities characterized by strong species diversity can better face challenging environment perturbations than those of low species diversity, thus conferring greater robustness to the ecosystem homeostasis (Degens et al. 2001). Associated changes in community structure are the net effect of individual members’ successful and unsuccessful acclimation to environmental perturbation. This shift in equilibrium is termed community adaptation (DeAngelis et al. 2010). It is now widely accepted that communities face short-term or fluctuating stress by acclimation (i.e., physiological adaptation), while continuous or predictable stress can be met by adaptation (i.e., genetically determined response) (Bradshaw and Hardwick 1989; Davison and Pearson 1996).

Although strong environmental disturbances are expected to alter the diversity of species (Rohr et al. 2006; Deng et al. 2007; Parnell et al. 2009), bacterial communities can mitigate the loss of species richness. For example, Tyson et al. (2004) showed that bacterial community adaptations in a highly impacted ecosystem (acid mine drainage [AMD]) involved several interacting metabolic networks between functionally differentiated species (Tyson et al. 2004). Recently, Lee et al. (2010) discovered a population-wide antibiotic resistance mechanism in *Escherichia coli* where few highly resistant mutants produce indole, a signaling molecule allowing more vulnerable genotypes to survive in stressful environmental conditions (Lee et al. 2010). More generally, several bacterial species can functionally collaborate, by exchanging small molecules, secreted by one species and metabolized by another, to ensure the survival of the whole community (Tyson et al. 2004; Harcombe 2010; Miller et al. 2010; Klitgord and Segrè 2011). Finally, many examples of horizontal gene transfers (HGT) have been reported as a major adaptive mechanism in natural bacterial communities (Shintani et al. 2008; Sobecky and Hazen 2009; Parnell et al. 2010, but see Zhaxybayeva and Doolittle 2011). Accordingly, two distantly related species could potentially occupy the same ecological niche, if one of them has transferred the relevant gene or group of genes to the other (Audic et al. 2007). The resultant decoupling between taxonomy and ecological function makes the use of a given species as a general bioindicator very problematic, especially when its identification is based on one gene (e.g., 16S rRNA). Instead, this is the functional interaction network of the whole community that will buffer environmental perturbations. Due to putative horizontal gene transfers, equivalent functional interaction networks may theoretically involve different species across similar ecosystems. However, HGT frequency is expected to decrease with genetic divergence between interacting species (Andam and Gogarten 2011). Therefore, two independent bacterial communities living in similar environmental conditions would exhibit the same overall taxonomical structure (i.e., members in both communities belonging to the same high taxonomic ranks). Consequently, ecotoxicologists should consider taxonomical structure at a community, rather than individual level, when predicting the response of bacterial communities to anthropogenic contaminants. Thus, comparing the overall taxonomic structures of bacteria communities facing a given environmental perturbation with reference communities is an invaluable experimental strategy to assess the health status of a given ecosystem.

Polymerase chain reaction-denaturing gradient gel electrophoresis (PCR-DGGE) is a cost-effective molecular technique employed as a culture-independent approach to investigate taxonomic structure and/or functional diversity in bacterial communities, depending on which kind of gene is under study. Classically, the 16S rRNA gene is targeted to profile taxonomic structure of bacterial communities, because it is a very conserved genomic region that also includes variable regions. Due to the inherent limitation of the method to achieve an unequivocal identification of single ribosomal sequence (or ribotype) (Ercolini et al. 2001), each band of the DGGE profile will be referred to an operational taxonomic unit (OTU) rather than a distinct species. Here, 16S-rDNA DGGE profiling of both environmentally disturbed and reference bacterial communities coupled with correlation analyses with abiotic factors will allow to rapidly measure the impact of the perturbation. Such an approach permits to identify the impact of environmental perturbation on parameters such as OTU richness, evenness, or dominance (see Methods).

Anthropological activities such as mining and smelting release toxic heavy metals such as arsenic (As), cadmium (Cd), lead (Pb), and copper (Cu). They impose a very strong selective pressure on the soil bacterial communities, causing structural changes in the community itself and are usually accompanied by a reduction in microbial biomass (Kandeler et al. 2000) and loss of biodiversity (Deng et al. 2007). Moreover, lacustrine communities under heavy radionuclide stress typically show higher structural similarities between each other when compared to communities from unpolluted lakes (Fields et al. 2005). These observations suggest that parallel community adaptations (sensu DeAngelis et al. 2010) could potentially be characterized. Given that evolutionary convergence represents one of the best illustrations of natural selection acting on the evolutionary trajectories of organisms (Derome and Bernatchez 2006), the present experimental approach focuses on several lacustrine ecosystems impacted by a similar and continuous environmental perturbation. Studying patterns of convergent or parallel evolution is a powerful mean to identify without a priori parameters that are truly associated to the selective pressure (Schluter 2000; Derome et al. 2006).

**Figure 1:**
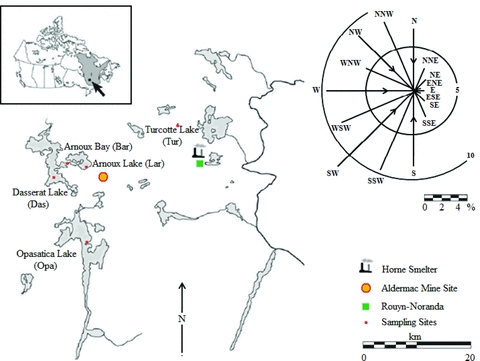
Geographical localization of Rouyn-Noranda (Abitibi-Temiscamingue, Canada) and the sampling sites visited in June 2010. Polluted reference site = Turcotte Lake, Clean reference site = Opasatica Lake, Test lake system = Arnoux Lake, Arnoux Bay, and Dasserat Lake.

Given that the physiological status of an ecosystem can be assessed by measuring the changes in the autochthonous microbial community in response to a disturbance (Bensal et al. 2011), the present study assess the accuracy of the PCR-DGGE technique, a valuable tool in microbial ecology, to monitor structural changes occurring in bacterial communities. The main goal of this study was to characterize the impact of heavy metals contamination on the taxonomic structure of lacustrine bacterial communities from five sites located in the surroundings of Rouyn-Noranda, Canada (Fig. 1). Among these five lakes, Opasatica Lake (Opa) was chosen as an unpolluted reference while Turcotte Lake (Tur) served as a positive control for pollution. The three other sites, namely Arnoux Lake (Lar), Arnoux Bay (Bar), and Dasserat Lake (Das), form a complex system of connected lakes exposed to a gradient of contaminants originating from a tributary of Arnoux Lake. This natural complex system is invaluable to characterize the influence of a continuous environmental stress on both independent and interconnected bacterial communities.

Two key questions in microbial ecology were addressed. First, we assessed whether a similar environmental stress has driven parallel changes at the taxonomic level on two independent bacterial communities. Second, we tested whether a long-term exposure to contaminants has induced significant taxonomic structure changes within the three interconnected bacterial communities. Furthermore, we tested whether similar OTU networks were correlated to a similar heavy metal contamination. To our knowledge, the parallel changes of natural lacustrine bacterial communities facing a similar environmental stress have never been studied before. The present study aims to generate meaningful insights on microbial community adaptation at the taxonomic level, under natural environmental conditions.

## Material and Methods

### Study area and sampling sites

The Rouyn-Noranda area (Quebec, Canada) has experienced strong mining activity over the last 50 years, generating polymetallic contaminant gradients (Couillard et al. 1993; Giguère et al. 2003, 2006). Located 15 km west of Rouyn-Noranda and 3 km northeast of Arntfield, Aldermac mine site is a 76 hectares area whose tailings consist of approximately 50% sulfurous minerals with significant concentrations of arsenic, cadmium, copper, molybdenum, zinc, and sulfur. As a result, areas adjacent to the mine tailings are strongly affected by AMD.

Five lakes were chosen in the surroundings of Rouyn-Noranda (see Fig. 1), following their relative metallic contamination combined with their geographical situation. Opasatica Lake (Opa) was chosen as an unpolluted lake reference, while Turcotte Lake (Tur) was chosen as a contaminated reference. The interconnected sites studied were Dasserat Lake (Das), Arnoux Bay (Bar), and Arnoux Lake (Lar). Through their interconnection, the water flow spreads the AMD from Arnoux Lake to Dasserat Lake such that a polymetallic gradient is generated.

### Sampling and filtration procedures

Sampling was undertaken during June 2010 by collecting 6 L of water at approximately 60 cm depth in the water column. Water samples were filtered first with a 3.0-µm mesh size, followed by a 0.22-µm nitrocellulose membrane (Advantec) using a peristaltic filtration pump (Masterflex L/S Pump System with Easy-Load II Pump Head, Cole-Parmer). Duplicates from the sampled lakes were placed into cryotubes containing 1 mL of sterile lysis buffer (40 mM EDTA, 50 mM Tris-HCl, 0.75 M sucrose) and then flash frozen in liquid nitrogen until DNA extraction.

### Abiotic sampling and survey

For each lake, pH and temperature were measured. Water samples were also collected in duplicates to determine trace metals (Al, Cd, Cu, Fe, Mn, Pb, Zn), major cations (Ca, Mg, Na, K, S), and dissolved organic carbon (DOC) at approximately 60 cm of depth in the water column. Water samples for determination of trace metals and major cations were taken with preconditioned syringes and processed through an Acrodisc Filter (VWR) into a 15-mL flask containing 300 µl of HNO_3_ (final concentration of 2%). All the samples were kept at 4°C before being analyzed at the INRS (Institut National de la Recherche Scientifique, Quebec) using ICP VISTA Varian-axial mass spectrometer. Samples for determination of the DOC were taken with sterile 50-mL scintillation vials and also analyzed at the INRS using a Total Organic Carbon (TOC) analyser Shimadzu VCPH with the Non-Purgeable Orgnic Carbon (NPOC) method (curve 0–5) and a detection limit set to 0.5 mg/L. Both sample replicates were processed to optimize measures of abiotic factors.

### DNA extraction

Genomic DNA was extracted using a modified protocol of salt extraction from Aljanabi and Martinez (1997) with the addition of a lysis step using lysozyme (Invitrogen) (1 mg/mL final concentration) and the pellet resuspended in 25 µl of sterile Milli-Q water. Subsequently, DNA integrity and quantity was controlled using a Nanodrop instrument (ND-1000, Nanodrop).

### Denaturing gradient gel electrophoresis (DGGE)

PCR-DGGE involves the electrophoretic separation of PCR amplicons in a polyacrylamide gel containing a gradient of chemical denaturants (urea and formamide). As the DNA molecule encounters an appropriate denaturant concentration, a sequence-dependent, partial denaturation of the double strand occurs and causes a reduced migration rate of the molecule. Every single band visible in DGGE represents a component of the bacterial community, whereas the number of bands reflects the community complexity.

PCR-DGGE targeting the V3–V5 regions of the 16S rRNA gene (Chakravorty et al. 2007) was realized using the universal bacterial primers 907R 5′-CCGTCAATTCMTTTRAGTTT-3′ (Lane et al. 1985) and 358F 5′-TACGGGAGGCAGCAG-3′ (Muyzer et al. 1993) with a GC-rich clamp at its 5′ end (5′-CGCCCGCCGCGC GCGGCGGGCGGGGCGGGGGCACGGGGGG-3′, Rölleke et al. 1998). Reactions were carried out for both duplicates in a volume of 16 µl containing 10 ng of template, 200 µM (each) dNTP (Promega), 0.3 µM/primer, 400 ng/µL BSA, 1.25 mM MgCl_2_, 1× Buffer, and 0.4U *Taq* DNA polymerase (Promega). PCR reactions were carried out in a Biometra T1 Thermocycler. The cycling program was 94°C for 5 min; 28 cycles of 94°C for 1 min, 52°C for 1 min, and 72°C for 3 min; and 72°C for 10 min. Both lake sample duplicates were run on a single gel. DGGE was performed using the D-Code universal mutation detection system (Bio-Rad). A total of 600 ng of PCR products were resolved on 10% (w/v) polyacrylamide gels at 40–60% denaturant gradient for 20 h at 60°C in 1× TAE and a constant voltage of 80 V. Gels were stained with SYBR Green 1 (Sigma) and visualized using a UV transilluminator (Vilmert-Lourmat).

### DGGE banding pattern analysis

The digitized image was imported into GelCompar II software (Applied Maths, Kortrijk, Belgium) and each band was considered an OTU. Bands visually common among all the sampled lakes were used as internal reference markers to normalize the fingerprint.

The intensities (*n_i_*) of the individual bands were first measured by GelCompar II software. Each band's relative intensity (*P_i_*) was then calculated as *P_i_* = *n_i_*/*N*, where *n_i_* is the densitometric curve intensity of band *i*, and *N* is the sum of the intensities for all the bands within the profile. Richness (*S*) was estimated simply as the number of bands detected in a single lane. The relative proportion of each band type was calculated using the number of representatives for a type of band over the richness in a lane. Each band-type contribution in a sample profile was calculated by means of the summation of all the intensities of a band type over the sum of the intensities for all the band types within a lane. The community biodiversity was established using three indices: (1) the Shannon Index (*H′* = –Σ(*P_i_* ln(*P_i_*)) (Shannon and Weaver 1963); (2) Simpson's Dominance Index (*c* = Σ(*P_i_*)^2^) (Simpson 1949); and (3) Pielou's Evenness Index (*J′* = *H*/ln*S*) (Pielou 1966). The homology between samples banding patterns, namely the beta diversity between two lakes, was calculated by means of Sørenson's Similarity Index (β) with the formula β = 2*C*/(*S_1_*+*S_2_*), where *S_1_* is the total number of species recorded in the first community, *S_2_* is the total number of species recorded in the second community, and *C* is the number of species common to both communities.

The GelCompar II software was set at 0.5% optimization and 0.5% position tolerance to calculate the Dice coefficient of similarity (Dice 1945). Cluster analyses were performed on the similarity matrix using the unweighted pair group method with arithmetic means algorithm (UPGMA), resulting in a dendrogram that graphically displayed the similarities among samples. Robustness analysis of the dendrogram topology was assessed using the “Cophenetic correlation” option, which computes a measure of clustering pattern consistency (values ranging from 0 to 100, 100 being the maximum consistency value). Comparing both community sample replicates further assessed the robustness of the dendrogram topology.

### Statistical analyses

All statistical analyses were performed using R statistical software (http://www.r-project.org/). To assess normality of the dataset, a Student *t*-test was first undertaken. ANOVAs were conducted using the R commands “model = lm(tableau$X∼tableau$Y)” and “anova(model).” The statistical model for the correlation analyses uses the values calculated from the DGGE fingerprint (X = Shannon, dominance, evenness, richness, beta diversity, proportion of each band type, contribution of each band type, intensities of the shared OTUs [5, 15, 17, 18, 21], intensities of the pollution-related OTUs [3, 10, 25, 44], intensities of the clean-related OTUs [16, 36, 39, 40, 42]) and environmental data collected on the field (Y = trace metals [Al, Cd, Cu, Fe, Mn, Pb, Zn] and major cations [Ca, Mg, Na, K, S], DOC, pH, and temperature).

Welch two-sample *t*-tests were realized with each abiotic parameter measured on the field (trace metals, major cations, DOC, pH, and temperature) by regrouping in the first sample the abiotic values for the polluted sites Arnoux Lake (xLar), Arnoux Bay (xBar), and Turcotte Lake (xTur), and the second vector consisting in the abiotic measures taken at the unpolluted sites Opasatica (xOpa) and Dasserat lakes (xDas). Effects for the ANOVA and Welch *t*-tests were deemed significant at *P* < 0.05 and marginally significant at *P* = 0.05–0.10.

## Results

### Environmental data

Trace metals (Al, Cd, Cu, Fe, Mn, Pb, Zn), major cations (Ca, Mg, Na, K, S), DOC, pH, and temperature for the studied lakes are summarized in Table 1. The most widely represented trace metal in all five lakes was iron (mean = 1.18428 mg/L; SD = 2.11585 mg/L) followed by aluminum (mean = 0.31470 mg/L; SD = 0.37202 mg/L), zinc (mean 0.19390 mg/L; SD = 0.22819 mg/L), manganese (mean = 0.14883 mg/L; SD = 0,19593 mg/L), copper (mean = 0.01990 mg/L; SD = 0.01901 mg/L), lead (mean = 0.00304 mg/L; SD = 0.00008 mg/L), and cadmium (mean = 0.00059 mg/L; SD = 0.00038 mg/L). Within the major cations tested, sulfur showed the highest concentration values (mean = 8.91042 mg/L; SD = 8.6144096 mg/L), followed by calcium (mean = 7.09774 mg/L; SD = 3.0044 mg/L), magnesium (mean = 2.20747 mg/L; SD = 1.13099 mg/L), sodium (mean = 1.52820 mg/L; SD = 0.98765 mg/L), and potassium (mean = 0.52211 mg/L; SD = 0.27990 mg/L). Neutral pH values were measured in the Opasatica Lake and Dasserat Lake (pH 7.64 and 7.11, respectively), while the pH values of Turcotte Lake, Arnoux Bay, and Arnoux Lake reached the acidic level (pH 4.91, 4.69, and 3.77, respectively). In addition, the highest DOC values were measured in Opasatica and Dasserat lakes (7.7 mg/L and 7.2 mg/L, respectively), followed by Arnoux and Turcotte lakes (4.4 mg/L and 3.8 mg/L, respectively) and Arnoux Bay (1.8 mg/L). Finally, the water temperature measured at sampling sites ranges from 16.5°C to 19°C.

**Table 1:** Abiotic parameters measured at each sampling site. (Al = aluminum, Ca = calcium, Cd = cadmium, Cu = copper, Fe = iron, K = potassium; Mg = magnesium, Mn = manganese, Na = sodium; Pb = lead, S = sulfur, Zn = zinc, DOC = dissolved organic carbon)

		Detection limit	Lar	Bar	Das	Tur	Opa
Trace metals (mg/L)	Fe	0.002	4.939	0.705	0.110	0.097	0.070
	Al	0.001	0.930	0.405	0.052	0.113	0.074
	Zn	0.0007	0.5574	0.2703	0.0347	0.1063	<0.0007
	Mn	0.0001	0.4421	0.2598	0.0036	0.0368	0.0019
	Cu	0.0005	0.0507	0.0243	0.0075	0.0141	0.0029
	Pb	0.003	0.003	<0.003	<0.003	<0.003	<0.003
	Cd	0.0002	0.0010	0.0007	0.0002	0.0009	<0.0002
Major cations (mg/L)	S	0.02	22.03	13.16	5.10	2.33	1.93
	Ca	0.02	9.92	8.26	7.34	2.00	7.97
	Mg	0.002	3.490	2.675	1.987	0.434	2.452
	Na	0.01	1.26	1.24	1.21	0.68	3.24
	K	0.002	0.597	0.461	0.497	0.139	0.917
Others	DOC	0.5	4.4	1.8	7.2	3.8	7.7
	pH	0.05	3.77	4.69	7.11	4.91	7.64
	Temp.	0.5	19.0	17.5	17.0	17.0	16.5

### DGGE fingerprint

**Figure 2:**
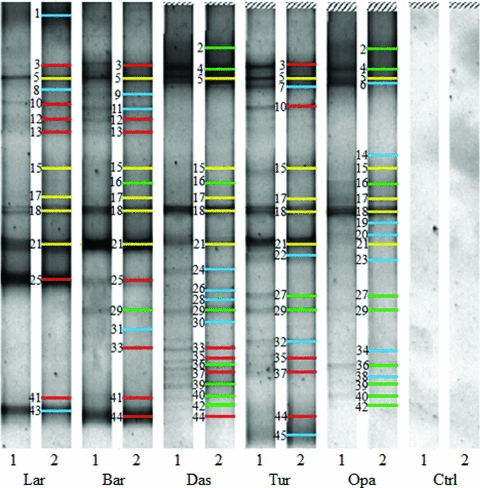
Denaturing gradient gel electrophoresis (DGGE) fingerprint. Bands in red are pollution-related operational taxonomic units (OTUs), green bands are OTUs shared with Opasatica Lake (undisturbed bacterial community reference), yellow bands represent OTUs common to all lakes, and blue bands indicate unique OTUs.

The DGGE profile for the overall experiment is showed on Figure 2. The highest species richness (*S*) was found in Dasserat and Opasatica Lakes (*S* = 21 for both) while Arnoux Lake, Arnoux Bay, and Turcotte showed weaker richness (*S* = 14, *S* = 17, and *S* = 16, respectively). Banding pattern analysis provided compelling evidence of five OTUs common to all sampling sites (OTUs 5, 15, 17, 18, and 21). Furthermore, Arnoux Lake, Arnoux Bay, and Turcotte Lake showed the highest number of pollution-related OTUs (i.e., OTUs present in at least two polluted lakes, but absent from the clean reference lake, *n* = 6, *n* = 7, and *n* = 5, respectively) and, in counterpart, the lowest number of clean-related OTUs (i.e., OTUs present in the clean reference lake, *n* = 0, *n* = 2, and *n* = 2, respectively). The inverse situation was observed in the case of Dasserat and Opasatica Lakes, which showed the highest number of clean-related OTUs (*n* = 8 and *n* = 9, respectively), and the lowest number of pollution-related OTUs (*n* = 4 and *n* = 0, respectively). By analyzing each lake's profile, three OTUs were found to be pollution specific (i.e., OTUs present at least in two polluted lakes, including the reference polluted lake, but excluding the clean reference lake) (OTUs 3, 10, and 44), while six OTUs were specific to cleaner lakes (i.e., OTUs present only in the two clean lakes, including the clean reference lake) (OTUs 2, 4, 36, 39, 40, and 42). Finally, several multiple unique OTUs were also present, with emphasis in the Opasatica Lake (*n* = 7). On the opposite, Turcotte and Dasserat lakes have lower number of unique OTUs (*n* = 4 for both) as for Arnoux Bay and Arnoux Lake (*n* = 3 for both).

**Figure 3:**
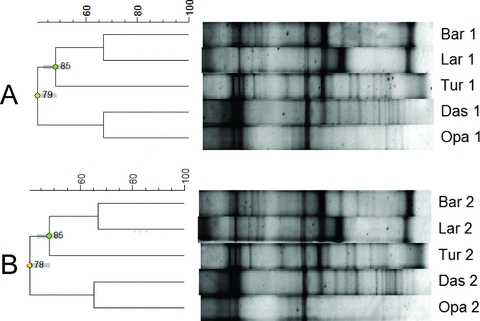
UPGMA clustering according to DGGE data using Dice coefficient of similarity. Values at the nodes in green and yellow represent cophenetic correlations (see Methods). (A) Community sample 1. (B) Community sample 2.

The homology between samples banding pattern was further analyzed for both duplicates using the dendrogram provided by GelCompar II software (Fig. 3). The dendrograms show two strongly differentiated clusters, the first one grouping Arnoux Lake, Arnoux Bay, and Turcotte Lake (mean = 85%, SD = 0% of cophenetic correlation) with 66.7% of similarity between the DGGE profile of the connected Arnoux Lake and Arnoux Bay, while the geographically isolated Turcotte Lake shares 48.0% of similarity with these two lakes. The second group visualized on the dendrogram encompasses geographically isolated Opasatica and Dasserat Lakes (78.5%, SD = 0.5% of cophenetic correlation), which share 65.9% (SD = 0.8%) of similarity together. Finally, Opasatica and Dasserat Lakes share only 25.1% (SD = 0.5%) of similarity with the polluted group (Arnoux Lake, Arnoux Bay, and Turcotte Lake. The high consistency of similarity and cophenetic values between both duplicates reflects the strong reproducibility of the experiment.

### Diversity and richness

The diversity and richness data collected for each sampling site are summarized in Table 2. Beta diversity values (β) indicate that the two interconnected lakes, Arnoux Lake and Arnoux Bay, were the most similar sampling sites visited (β = 0.645), while Dasserat Lake was more similar to Opasatica Lake (β = 0.585) than to Arnoux Bay (β = 0.486). The reference polluted Turcotte Lake exhibited similarity with both Arnoux Bay (β = 0.485) and Dasserat (β = 0.5) connected lakes. Then, the highest alpha diversity values were found in Dasserat and Turcotte Lakes (*H′* = 2.438 and *H′* = 2.408, respectively) followed by Opasatica Lake (*H′* = 2.313), Arnoux Lake (*H′* = 2.251), and Arnoux Bay (*H′* = 2.112). The highest evenness (*J′*) values were found in Turcotte and Arnoux Lakes (*J′* = 0.868 and *J′* = 0.853, respectively), followed by Dasserat Lake (*J′* = 0.801), Opasatica Lake (*J′* = 0.76), and Arnoux Bay (*J′* = 0.745). The highest dominance value is found in Arnoux Bay (*c* = 0.197), followed by Arnoux Lake (*c* = 0.140), Dasserat Lake (*c* = 0.126), Opasatica Lake (*c* = 0.124), and Turcotte Lake (*c* = 0.114).

**Table 2:** Alpha diversity, evenness, richness, dominance, and beta diversity calculated for the studied system. (*H′* = Shannon Index, *J′* = Pielou's Evenness Index, *S* = Specific Richness, *c* = Simpson's Dominance Index)

	Alpha diversity	Evenness	Richness	Dominance	Beta diversity				
					
	*H′*	*J′*	*S*	*c*	Lar	Bar	Das	Tur	Opa
Lar	2.251	0.853	14	0.140	1.000	0.645	0.294	0.467	0.286
Bar	2.112	0.745	17	0.197	–	1.000	0.486	0.485	0.368
Das	2.438	0.801	21	0.126	–	–	1.000	0.500	0.585
Tur	2.408	0.868	16	0.114	–	–	–	1.000	0.378
Opa	2.313	0.760	21	0.124	–	–	–	–	1.000

### Correlation analyses

Welch two-sample *t*-tests put in evidence significant differences between polluted lakes versus clean lakes concerning cadmium (*P* = 0.02086), DOC concentrations (*P* = 0.02633), and pH measures (*P* = 0.006393).

The species richness showed significant correlations with cadmium gradient (negative correlation, *R*^2^ = 0.97; *P* = 0.002396) and pH values (*R*^2^ = 0.96; *P* = 0.003967). Furthermore, significant correlations between beta diversity in Arnoux Lake and multiple heavy metals (Zn: *R*^2^ = 0.99; *P* = 0.000409, Cu: *R*^2^ = 0.99; *P* = 0.001029, Al: *R*^2^ = 0.96; *P* = 0.003678, Mn: *R*^2^ = 0.96; *P* = 0.003809, Fe: *R*^2^ = 0.84; *P* = 0.02871), sulfur (*R*^2^ = 0.89; *P* = 0.01533) and pH (negative correlation, *R*^2^ = 0.81; *P* = 0.03863) were found, as was a significant influence of calcium over beta diversity in Turcotte Lake (negative correlation, *R*^2^ = 0.87; *P* = 0.02155). On the other side, the beta diversity of Opasatica Lake exhibits significant correlations with sodium (*R*^2^ = 0.81; *P* = 0.0363) and pH values (*R*^2^ = 0.82; *P* = 0.03374).

Statistical analyses also reveal that the proportion of shared bands was significantly correlated with DOC values (*R*^2^ = 0.88; *P* = 0.01761), while the proportion of pollution-related OTUs was influenced by pH (*R*^2^ = 0.82; *P* = 0.01594) and the proportion of clean-related OTUs was significantly related to iron and lead gradient (negative correlation for both, *R*^2^ = 0.89; *P* = 0.01638 and *R*^2^ = 0.93; *P* = 0.008178, respectively). Then, pollution-related OTUs exhibit significant correlations with both cadmium concentrations (*R*^2^ = 0.88; *P* = 0.01836) and pH values (negative correlation, *R*^2^ = 1.00; *P* = 3.972 × 10^–5^). Finally, clean-related OTUs also exhibit significant correlations to both cadmium (negative correlation, *R*^2^ = 0.89; *P* = 0.01689) and pH measures (*R*^2^ = 0.87; *P* = 0.02177).

Considering each band from the DGGE fingerprint, shared OTU 18 was influenced by pH (*R*^2^ = 0.85; *P* = 0.02681), and shared OTU 21 was significantly correlated with DOC concentrations (negative correlation, *R*^2^ = 0.90; *P* = 0.01444). Furthermore, OTU 25 was the only pollution-related species to significantly show correlation with multiple heavy metals (Fe: *R*^2^ = 1.00; *P* = 7.616 × 10^–6^, Mn: *R*^2^ = 0.79; *P* = 0.04488, Pb: *R*^2^ = 0.99; *P* = 4.2929 × 10^–4^, Al: *R*^2^ = 0.92; *P* = 0.01055, Cu: *R*^2^ = 0.88; *P* = 0.01809, and Zn: *R*^2^ = 0.86; *P* = 0.02275, S: *R*^2^ = 0.81; *P* = 0.03856), and with the temperature (*R*^2^ = 0.91; *P* = 0.01123). Then, shared OTU 17 showed correlation with sodium (*R*^2^ = 0.96; *P* = 0.003479). Finally, OTU 16 is the sole clean-related OTU to significantly exhibit correlation with sodium concentrations (*R*^2^ = 0.90; *P* = 0.01591).

Aside from the above significant correlations, multiple marginally significant correlations (*P* = 0.05–0.10) were found using ANOVAs, especially considering the cadmium and DOC parameters. Indeed, cadmium was marginally correlated with *i* beta diversity in Opasatica Lake (negative correlation, *R*^2^ = 0.68; *P* = 0.08336), *ii* the proportion of pollution-related OTUs (*R*^2^ = 0.68; *P* = 0.08772), *iii* the shared OTU 18 (negative correlation, *R*^2^ = 0.69; *P* = 0.08309), *iv* the pollution-related OTU 10 (*R*^2^ = 0.69; *P* = 0.07967), and *v* the clean-related OTUs 36 and 40 (negative correlation for both, *R*^2^ = 0.70; *P* = 0.07868 and *R*^2^ = 0.67; *P* = 0.08636, respectively). Then, DOC showed marginal significant correlations with *i* the interlake “beta diversity” of Arnoux Bay (negative correlation, *R*^2^ = 0.72; *P* = 0.07071), *ii* proportion of pollution-related OTUs (negative correlation, *R*^2^ = 0.75; *P* = 0.056), *iii* contribution of pollution-related OTUs (*R*^2^ = 0.70; *P* = 0.07936), and *iv* the intensity of OTU 18 (*R*^2^ = 0.74; *P* = 0.05962).

Finally, the Shannon, evenness, and dominance indexes were not correlated with any of the environmental parameters.

## Discussion

As anthropogenic activities increasingly deteriorate natural ecosystems, there is an urgent need to elucidate how microbial communities acclimate or adapt to buffer such environmental stress. Such insights aid the identification key microbial consortia and assist in the development of durable mitigating strategies to restore impacted ecosystems. Here, we tested the power of resolution of the PCR-DGGE technique as a cost-effective molecular method to rapidly assess the level of perturbation of five lacustrine ecosystems exposed to contrasting concentrations of heavy metals. The underlying hypothesis was that the overall taxonomic structure of a microbial community is mirroring the health status of its ecosystem (Rohr et al. 2006; Deng et al. 2007; Parnell et al. 2009).

Therefore, the objective of this survey was threefold. First, to assess whether a similar anthropogenic pressure drives parallel changes at the taxonomic level among two geographically isolated bacterial communities. Second, to test whether long-term exposure to heavy metals, which generated a contamination gradient for three interconnected lacustrine bacterial communities, has driven significant taxonomic structure changes. Finally, the third objective was to assess whether PCR-DGGE has sufficient resolution to unambiguously characterize the signature of environmental perturbation on the taxonomical structure of natural bacterial communities.

In the following sections, the correlation between DGGE profiles and abiotic factors will be examined to assess their respective influences on the taxonomic structure of the five bacterial communities.

Geochemical analyses of lake water samples indicate a decrease in heavy metal concentrations within the interconnected sites of Arnoux Lake, Arnoux Bay, and Dasserat Lake, creating a natural polymetallic gradient. Concentrations of all heavy metals are highest in Arnoux Lake, decrease slightly in Arnoux Bay, and drop substantially in Dasserat Lake. This phenomenon is the consequence of a strong dilution factor between Arnoux Bay and Dasserat Lake. As a result, heavy metals’ concentrations in Opasatica were similar to those found in Dasserat Lake, while heavy metals’ concentrations in Turcotte Lake were similar to both Arnoux Bay and Arnoux Lake. Correlatively, the dendrogram topology obtained from the PCR-DGGE fingerprint statistically supports grouping bacterial communities from two independent clean sites, namely Opasatica and Dasserat lakes on one side, and from two independent polluted sites, namely the connected Arnoux Lake–Arnoux Bay system and Turcotte Lake, on the other side. Taken together, these results first suggest that a strong abiotic perturbation can drive significant shifts in the taxonomic structure of bacterial communities from interconnected lakes following a gradient of contaminants, and furthermore, that two geographically isolated bacterial consortia can undergo parallel community adaptation if impacted by a similar environmental pressure.

**Figure 4:**
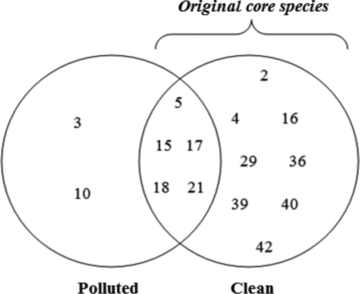
Venn diagram representing the main taxonomical relationships between bacterial communities from polluted and clean lakes. Each number represents an OTU.

The five studied lakes are located on the same glaciolacustrine deposits left by the postglacial lake Barlow-Ojibway, 10,000 years ago (surficial geology map No. 1639A [Geol. Surv. Can.; Energy, Mines and Resources Canada]). By triggering major founder events, the post-Pleistocene ice retreat has been identified as a key factor in shaping present-day genetic structure of many species (Hewitt 1996, 2004), including aquatic organisms (Bailey and Smith 1981). Therefore, it suggests that the five OTUs shared between all the five lakes (OTUs 5, 15, 17, 18, and 21) can be considered as ancestral core OTUs, while the 13 OTUs shared between Opasatica and Dasserat lakes (OTUs 2, 4, 5, 15, 16, 17, 18, 21, 29, 36, 39, 40, and 42) plus the ancestral core very likely reflect the original core species of the communities from Arnoux Lake, Arnoux Bay, and Turcotte Lake before being submitted to the recent environmental disturbance (Fig. 4). Furthermore, our results show unambiguously that two similar heavy metal contaminations drove parallel taxonomical changes of independent communities. More precisely, two pollution-specific OTUs (OTUs 3 and 10) were enriched independently in Turcotte Lake and in the connected Arnoux Lake–Arnoux Bay system. These OTUs represent the heavy metals resistant core OTUs, which acclimated better and/or first developed resistance mechanisms, thus allowing them to reach higher abundance in contaminated lakes. These heavy metals resistant core OTUs were likely present in all communities at very low abundance before the mining activities started, and increased gradually in abundance as the level of metal contamination increased. Indeed, some taxonomic groups are naturally less sensitive to heavy metal stress than others. This is particularly the case of γ-proteobacteria and β-proteobacteria, those were observed to represent 64% of the bacterial diversity of a heavy metal contaminated ground water microbial community (Hemme et al. 2010). From now, three nonexclusive hypotheses have to be explored to establish why other members of the bacterial community showed different survival success across the three polluted lakes (i.e., occurrence of lake specific OTUs).

The first hypothesis of such a discrepancy can be the loss of strains due to genetic drift, and/or variable capacity of acclimation among closely related strains across the three polluted lakes. This phenomenon was observed in *Drosophila* where populations originating from neighboring localities exhibited differential capacity of acclimation to stress (Sarup and Loeschcke 2010). However, it is more likely that continuous exposure to a strong environmental stress was met by a genetically determined response (Bradshaw and Hardwick, 1989; Davison and Pearson 1996). Therefore, the second hypothesis implies conjugative plasmid transfer, the most important mechanism by which bacterial genetic material can be exchanged to cope with rapidly changing environmental conditions (Torsvik et al. 2002; Grohmann 2011). Furthermore, heavy metal tolerance mechanisms adapted in bacteria are often plasmid borne, and therefore capable of being spread throughout a bacterial community by HGT (Coombs and Barkay 2004; Martinez et al. 2006). HGT mediated by plasmid transfer have been documented in natural conditions for lake water (Shintani et al. 2008; Parnell et al. 2010) and seawater (Sobecky and Hazen 2009) bacterial communities. Under this scenario, the heavy metals resistant core OTUs may have transferred resistance genes to both the lake-specific OTUs and the ancestral core OTUs (species shared in all five lakes). Interestingly, the occurrence of lake-specific OTUs suggests that different OTUs disappeared in the three polluted lakes. Such a discrepancy can be explained by (1) loss of strains due to genetic drift, and/or (2) maladaptation due to different HGT network dynamics across lakes. Connectivity, that is functional compatibility, between OTUs is a statistically significant factor in determining HGT efficiency (Cohen et al. 2011). Then, bacteria are more prone to HGT in biofilms than in open water because HGT efficiency is mainly constrained by cell density (Van Elsas and Bailey 2002). Therefore, HGT network dynamics in open lake water would mainly depend on the contingency of one or another functionally compatible OTUs in the direct vicinity of the HGT donor, thus driving in turn the occurrence of lake-specific OTUs. The third hypothesis explaining how resistance mechanisms were transferred to other members of the bacterial community does not invoke any genetic changes to the community. Lee et al. (2010) demonstrated that few highly resistant mutants provided a population-wide resistance by producing indole, a signaling molecule allowing more vulnerable genotypes to survive in stressful environmental conditions. As cadmium is known to bind thiol groups, leading to glutathione depletion and oxidation of sulfhydryl groups in proteins (Lourenço and Gomes 2009), the increase in the intracellular cadmium concentration might be mitigated by the increased production of glutathione from the heavy metal resistant core OTUs.

To sum up, parallel community modifications resulting from a similar environmental perturbation may have occurred due to selection of a heavy metal resistant core OTUs combined to the loss of most sensitive bacterial species. Then, differences observed between the three polluted lakes may result from genetic drift and/or differential networks of horizontal transfers of resistance genes. Alternatively, the heavy metal resistant core OTUs may provide a population-wide resistance mechanism by producing signaling molecules mitigating the physiological impact of heavy metal contamination.

The significant correlations found with ANOVA between environmental parameters and biodiversity indices strongly suggest that pH and cadmium exert the widest spectral influence over the taxonomic structure of the bacterial communities. Taking into consideration its numerous marginal correlations, the DOC parameter could be considered as a secondary key environmental measure. Interestingly, Correlation analyses suggest that pH and cadmium share a similar spectral influence, as with the DOC parameter to some extent. pH reduction is also known to induce a decline in the bacterial structural and functional diversity in other systems (Anderson et al. 2009), and cadmium is the most harmful heavy metal for microorganisms (Nies 1999). However, low pH values have been widely reported to have a protective effect on metal toxicity in a variety of organisms including bacteria (Franklin et al. 2000; Sandrin and Maier 2002; Worden et al. 2009). Consequently, the significant correlation between cadmium gradient and pH values collected reveals that cadmium still has a strong impact over the structure and the functionality of a bacterial community, despite of water acidity. Therefore, cadmium concentration is an abiotic factor of major concern for evaluating the buffering potential of a lacustrine ecosystem.

In conclusion, our results bring to light significant evidence that similar environmental perturbations drive parallel changes at the taxonomic level on two independent bacterial communities. Then, fluctuations taking places in the dominant microbial populations under a polymetallic gradient were characterized. More precisely, correlation analyses between OTUs and abiotic factors allowed identifying physicochemical parameters that have the strongest influence on the whole community network. Such insights therefore clearly demonstrate the need to study bacterial community structural changes triggered by abiotic contaminant exposure under an ecological and evolutionary points of view. Indeed, further analyses will allow detecting community members that are functionally interdependent and adapted to cope with stressing abiotic factors. Finally, this study demonstrates that using a low-cost molecular tool such as PCR-DGGE is very suitable to assess the health status of lacustrine ecosystems by profiling the taxonomic structure of bacterial communities. By allowing to monitor a dozen of bacterial community samples per gel twice a day, this technique has still undoubtedly a great value. PCR-DGGE was recently applied to monitor various types of microbial communities, such as root-associated microbiota (Kolton et al. 2011), gut microbiota (Wang et al. 2011), coral mucus microbiota (Meron et al. 2011), forest soil (Kauppi et al. 2011), bioreactors (Vejmelkova et al. 2011), or even agriculture products (Aquilanti et al. 2011). Furthermore, PCR-DGGE profiling is a valuable tool that allows screening various community samples to target those exhibiting the most contrasted pattern for further investigations. Using classical molecular methods, the next steps to characterize further the taxonomic structure of bacterial communities are labor and time consuming. First, sequencing every band of the DGGE profiles to identify the 16S ribotype requires as many cloning steps, because many bands contain several closely related ribotypes. Then, quantifying the relative abundance of every ribotype necessitates to develop as many pairs of real-time PCR primers. Knowing that lacustrine bacterial communities encompass several hundreds of ribotypes (K. Laplante et al., unpubl. data), accurately characterizing biodiversity by cloning approach is not cost effective. The very recent development of both next-generation sequencing and bioinformatics will now permit to characterize in a single step both the taxonomic diversity and the relative abundance of every ribotype. Furthermore, high throughput sequencing of metatranscriptomic libraries will provide further insights into characterizing the dynamic behavior of the more active members of the bacterial communities, both at functional and taxonomic levels. These invaluable tools will allow researchers to explore further the functional network adaptability of bacterial communities, and in turn, to predict their capacity to maintain ecosystem homeostasis when facing rapid environmental changes. Such investigations on the lake system presented here are currently underway.
